# Toxic Effects of Tartrazine and the Protective Role of Curcumin on Liver Function and DNA Integrity in Male Rats

**DOI:** 10.1002/fsn3.71213

**Published:** 2025-12-16

**Authors:** Metin Varlı, Mehmet Cihan Yavaş, Fazile Cantürk Tan, Kardelen Tur, Güldidar Basmacı

**Affiliations:** ^1^ Department of General Surgery, Faculty of Medicine Mardin Artuklu University Mardin Turkey; ^2^ Department of Biophysics, Faculty of Medicine Mardin Artuklu University Mardin Turkey; ^3^ Department of Biophysics, Faculty of Medicine Erciyes University Kayseri Turkey; ^4^ Department of Pathology Ministry of Health Mardin Education and Training Hospital Mardin Turkey

**Keywords:** curcumine, food, liver, oxidative stress, tartrazine

## Abstract

Tartrazine (TAR) and curcumin (CUR) are commonly utilized in the food manufacturing sector. The present investigation was designed to assess the hepatotoxic impact of the food dye tartrazine on hepatic function and its related biomarkers. We systematically allocated 35 male Wistar rats into five homogeneous groups. The specified groups consisted of: control, TAR at a dosage of 10 mg/kg/day, TAR at a dosage of 100 mg/kg/day, TAR at a dosage of 10 mg/kg/day combined with CUR at 20 mg/kg/day, and TAR at a dosage of 100 mg/kg/day combined with CUR at 20 mg/kg/day. All experimental groups received the treatment via oral gavage. Our findings indicated that as the TAR dosage escalated relative to the control group, the levels of superoxide dismutase (SOD), aspartate aminotransferase (AST), alanine aminotransferase (ALT), lactate dehydrogenase (LDH), alkaline phosphatase (ALP), and gamma‐glutamyl transferase (GGT) exhibited an increase, while the alpha‐fetoprotein (AFP) level demonstrated a decline. In the CUR‐treated groups, in comparison with the control groups, the levels of SOD, AST, ALT, LDH, total bilirubin, GGT, and AFP increased in the low‐dose TAR groups, whereas ALP levels decreased. Our histopathological analysis disclosed the occurrence of degenerative changes in both TAR and CUR treatment groups. The genotoxic assessment, utilizing the DNA comet assay, revealed that an increase in TAR dosage corresponded with heightened DNA damage; however, the incorporation of CUR mitigated this detrimental effect. Our findings suggest that tartrazine may exert deleterious effects on hepatic function, whereas curcumin has displayed partial therapeutic efficacy.

## Graphical abstract

Tartrazine exposure led to dose‐dependent increases in liver enzyme biomarkers and DNA damage, while co‐administration of curcumin reduced some of these effects. Histopathological and biochemical findings suggest that curcumin provides partial protection against tartrazine‐induced liver injury and DNA damage.

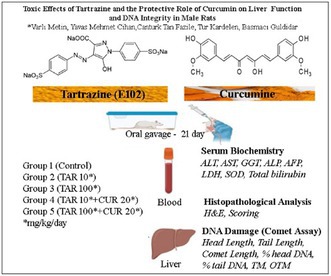

## 1 Introduction

Azo dyes, which are synthetic colorants characterized by their vivid color palettes, are extensively employed within the food, cosmetics, and textile sectors (Hanna et al. 2023; Pay et al. 2023). The production of beverages, especially sports drinks, frequently uses tartrazine, known as E102 in the European Union and FD&C Yellow 5 in the United States, due to its bright and attractive color (Kaya et al. 2021; Priscillal and Wang 2023). Organizations like the Food and Drug Administration, European Food Safety Authority (EFSA), and the World Health Organization (WHO) publish approval processes and regulations, stating that the acceptable daily intake of tartrazine is 0–10 mg/kg based on body weight (World Health Organization and Food and Agriculture Organization of the United Nations 2017; Al‐Daamy and Al‐Zubiady 2020; Altinoz et al. 2021; Abd Elaziz et al. 2024). The use of tartrazine in real life, particularly at low doses, raises serious concerns regarding individual exposure (Amchova et al. 2024).

Synthetic food colorings, such as sunset yellow and tartrazine, have significant genotoxicity and cytotoxicity effects on humans by increasing the level of DNA damage and the percentage of apoptosis. Researchers are also investigating the negative effects of synthetic food colorings on reactive oxygen species (ROS) (El‐Borm et al. 2020). There have been reports of harmful effects on consumer health from some artificial coloring additives. According to Hassan (2010), researchers are using the comet assay to investigate the genotoxic effects of tartrazine E102, a substance that damages DNA in the liver.

Tartrazine can have significant biochemical and histological effects on Wistar albino rats, and long‐term consumption can have toxic effects on human health (Abd Elaziz et al. 2024). Abd Elaziz et al. (2024) reported that tartrazine intake causes histological changes in liver tissue. Another study demonstrated a dose‐dependent increase in AST, ALT, LDH, urea, creatinine, SOD, AFP, GGT, and ALP, along with a dose‐dependent increase in DNA damage (DNA in tail). However, studies (El Golli 2016; El‐Desoky et al. 2022; Ibrahim et al. 2024) have demonstrated that the intake of curcumin (50 mg/kg) alleviated tartrazine‐induced toxicity.

The food industry enhances the appeal of various products, including food colorings, to reach the end consumer. This study investigated the impact of tartrazine exposure on liver tissue and its associated biochemical parameters, and assessed the effectiveness of curcumin, which has demonstrated protective effects against exposure, in this context. Additionally, in our study, we analyzed the histopathology of liver tissue and tissue‐associated parameters such as ALT, AST, ALP, LDH, GGT, total bilirubin, and AFP. This study identified the broadest potential damage tartrazine exposure could cause to the liver, while also investigating the protective effect of curcumin against potential toxicity.

## 2 Material and Methods

### 2.1 Experimental Model

The study included healthy (300–350 g) and 3‐month‐old male Wistar albino rats. The study began after the animals acclimatized in standard cages for 1 week. During a 12‐h light/dark cycle, they were fed standard pellet feed and tap water at a temperature of 22°C ± 2°C. We conducted the study in accordance with the Helsinki Declaration. The studies of Bastaki et al. (2017) determined the dosage limits of tartrazine and curcumin, while the studies of Essawy et al. (2023) determined the application duration. In addition, for low‐dose tartrazine, Amin and Abdel Hameid (2010) and for high‐dose, Abdelaziz et al. (2019) studies were also references for us. In the study of El‐Ashmawy et al. (2013), it contributed to our study because it was the value commonly chosen for curcumin. The 35 rats to be used in the study will be randomly divided into 5 groups, with 7 rats in each group. Group 1 was the control group and got no medicine for 21 days. Our study determined the duration of exposure because it was short‐term and close to the duration used in subacute studies OECD (2008). Group 2 got 10 mg/kg tartrazine (Sigma‐Aldrich Chemie GmbH Tartrazine CAS‐No: 1934–21–0) and 0.25 mL of distilled water mixed with tartrazine. Group 3 got 100 mg/kg tartrazine. Group 4 got 10 mg/kg tartrazine and 20 mg/kg curcumin (Sigma‐Aldrich Chemie GmbH *Curcuma longa* CAS‐No: 458–37‐7); and Group 5 got 100 mg/kg tartrazine and 20 mg/kg curcumin (Sigma‐Aldrich Chemie GmbH *Curcuma longa* CAS‐No: 458–37‐7).

### 2.2 Evaluation of DNA Damage

We used the comet approach in a neutral environment to assess DNA damage in liver tissue. We used a sterile scalpel to cut approximately 0.5 g of liver tissue. Once the procedure was complete, we performed ethidium bromide staining. A fluorescent microscope (Olympus, BX51, Tokyo, Japan) set to 200× magnification was used to acquire measurements. We used the Comet Assay Software Project (CASP‐1.2.2, Windows 2010) to photograph 100 randomly selected cells, capturing seven metrics: comet length, head length, tail length, tail moment (TM), olive tail moment (OTM), and the percentage of DNA in the head and tail. All other cell heads were considered unharmed; however, the “comet” pattern created by moving DNA fragments from the cell head suggested damage (Akdag et al. 2016; Cantürk Tan et al. 2020; Yavaş et al. 2024).

### 2.3 Histopathological Analysis

We prepared paraffin blocks from liver specimens after formalin fixation. We obtained sections with a thickness of 5 μm for evaluation under a light microscope. We stained the sections with hematoxylin and eosin. A light microscope conducted the histopathological evaluation, examining hepatocytes, portal areas, and vascular structures. We scored portal inflammation as follows: “score 0: none, score 1: mild, present in some areas, score 2: moderate, present in most areas, score 3: moderate/marked, present in all areas, score 4: marked, present in all areas.” We scored interface hepatitis as follows: “score 0: none, score 1: mild, focal presence, score 2: mild/moderate, focal presence, score 3: moderate, continuous but present in less than 50% of the portal area, score 4: severe, continuous and present in more than 50% of the portal area.” We scored lobular inflammation as follows: “score 0: none, score 1: 1 or fewer foci per 10 HPF, score 2: 2‐4 foci per 10 HPF, score 3: 5‐10 foci per 10 HPF, score 4: more than 10 foci per 10 HPF.” The presence of apoptosis in hepatocytes was evaluated as follows: “score 1: < 1 apoptosis/40 BBA, score 2: 1‐3 apoptosis/40 BBA, score 3: > 3 apoptosis/40 BBA.” We evaluated and scored the presence of focal (spotty) necrosis in hepatocytes as follows: “score 0: none, score 1: 1 or fewer foci per 10 BBA, score 2: 2‐4 foci per 10 BBA, score 3: 5‐10 foci per 10 BBA, score 5: more than 10 foci per 10 BBA.” We evaluated and scored the presence of mitosis in hepatocytes as follows: “score 0: none, score 1: present, focal, score 2: present, diffuse.” We evaluated the presence of microvesicular steatosis in hepatocytes and calculated its ratio to all hepatocytes if present. We evaluated the presence of eosinophils in the portal area, hepatocyte ballooning, Mallory‐Denk bodies, bile duct epithelium damage, ductular proliferation, ductular reaction, bilirubin stasis, cholestasis, sinusoidal dilation, and venous congestion (Yavaş and Kilitci 2023).

### 2.4 Serum Biochemistry Levels for Rats

Under anesthesia, blood samples were taken from rats via the intracardiac route and centrifuged at 4500 rpm for 5 min (elektromag M815A device). Then the analysis started. Alkaline phosphatase (ALP) (cat no: OttoBC124), aspartate aminotransferase (AST) (cat no: OttoBC127), alanine aminotransferase (ALT) (cat no: OttoBC128), lactate dehydrogenase (LDH) (cat no: OttoBC129), total bilirubin (cat no: OttoBC132), and gamma‐glutamyl transferase (GGT) (cat no: OttoBC141) levels were measured using a colorimetric method using the Mindray‐BS400 model fully automatic biochemistry device (Otto Scientific, Shanghai, China). Colorimetric analyses were continued according to the standard method. Rat alpha‐fetoprotein (AFP) (cat no: E0332Ra, BT Lab, China) levels were measured using the microelisa method using the Biobase EL10C model fully automatic biochemistry device (Kılınç et al. 2023; El‐Zehery et al. 2024; Cetik Yildiz et al. 2024).

### 2.5 Statistical Analysis

We classified the DNA comet results and biochemical data as quantitative and categorical variables according to the groups in our study. To assess the distribution of the data, we used the SPSS 26.0 software for Windows. One‐way ANOVA was performed for group comparisons, and the Student's *t*‐test was used for pairwise comparisons. A *p*‐value of less than 0.05 was considered statistically significant.

## 3 Results

SOD and ALP concentrations increased in the TAR 10 and TAR 100 groups compared to the control group, and curcumin supplementation caused a decrease (*p* > 0.05). When compared to the control group, the ALP concentration went up in the TAR 10 and TAR 100 groups, but it went down when curcumin was added (*p* = 0.026, *p* < 0.05). It was seen that the concentration level went up a lot in the AST group as the tartrazine dose went up, but it went down in the group where curcumin was added to the medium (*p* = 0.024, *p* < 0.05). In the comparison with the control group, we observed a decrease in ALT and LDH in the low‐dose tartrazine group, an increase in serum concentration in the high‐dose tartrazine group, and a subsequent decrease in the group receiving both high‐dose tartrazine and curcumin (*p* > 0.05). In the statistical comparison with the control group, we observed an increase in the serum concentration levels of total bilirubin, GGT, and AFP in the low‐dose tartrazine group (TAR 10 mg/kg), a decrease in total bilirubin and AFP, and a further increase in GGT in the high‐dose group (TAR 100 mg/kg). Table 1 presents all results (*p* > 0.05).

**TABLE 1:** Effects of Tartrazine (TAR) and Curcumin (CUR) on liver serum biochemical functions in male albino rats.

Parameter	Control	TAR‐10 mg	TAR‐100 mg	TAR‐10 + CUR‐20 mg	TAR‐100 + CUR‐20 mg	*p*
SOD (U/mL)	4.14 ± 0.27	4.78 ± 2.05	4.88 ± 0.61	4.53 ± 0.39	4.81 ± 0.59	0.655
AST (U/L)	81.52 ± 17.77	79.55 ± 17.99	90.47 ± 25.42	113.00 ± 32.00^a^	72.55 ± 17.07^a^	0.024
ALT (U/L)	65.32 ± 16.78	62 ± 17.95	71.94 ± 18.03	88.51 ± 25.62	58.74 ± 17.14	0.768
LDH (U/L)	250 ± 153.17	240 ± 178.35	289 ± 164.21	290.00 ± 148.30	196.86 ± 111.23	0.768
Total Bilirubin (mg/dL)	0.29 ± 0.07	0.31 ± 0.02	0.29 ± 0.03	0.32 ± 0.05	0.29 ± 0.02	0.507
ALP (U/L)	356.29 ± 77.17	415.43 ± 80.53	583.7 ± 333.6^b^	330.86 ± 351.55	163.43 ± 61.89^b^	0.026
GGT (U/L)	1.83 ± 0.52	2.04 ± 0.95	3.69 ± 2.43	2.65 ± 1.03	1.80 ± 0.76	0.059
*AFP* (ng/mL)	0.15 ± 0.13	0.21 ± 0.03	0.10 ± 0.07	0.19 ± 0.08	0.16 ± 0.10	0.232

*Note:* Data are presented as mean ± standard deviation (SD). Statistical analysis was performed using ANOVA variance analysis. For comparisons between two groups, the Student's *t*‐test was applied (*p* < 0.05). Parameters with statistically significant differences between the two groups are indicated by the same letter (a or b).

**FIGURE 1:**
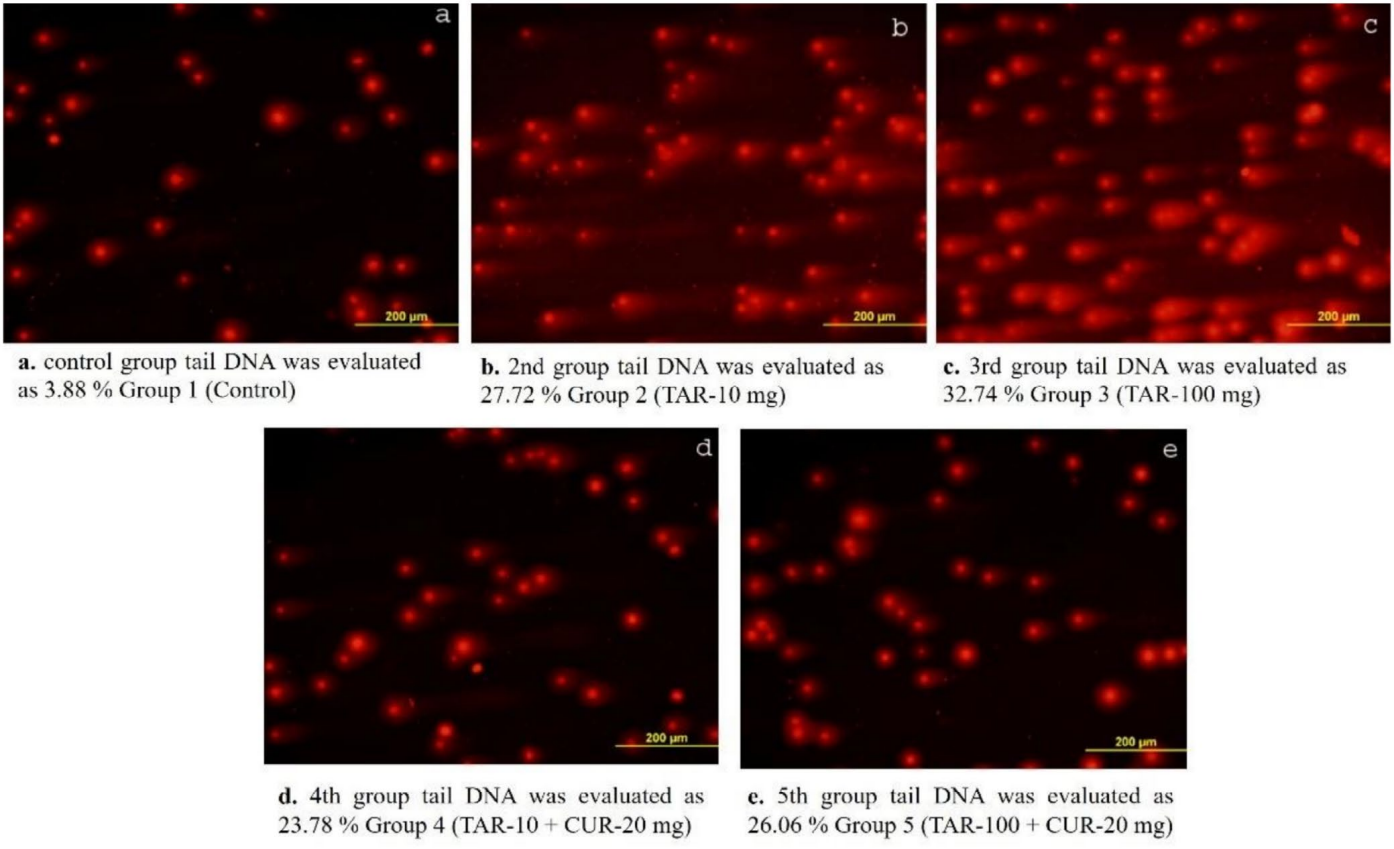
DNA damage in liver tissue assessed by comet assay in all experimental groups.

We evaluated liver tissues from each group using the alkaline comet assay protocol for DNA damage. We presented the evaluation using seven comet parameters: head length, tail length, comet length, percent head DNA, percent tail DNA, tail moment, and OTM (the olive tail moment). For these parameters, a statistically significant difference was detected among all groups (*p* < 0.001). To determine mechanical damage, the % tail DNA parameter in the control group was 3.88 ± 0.12, in the tartrazine 10 mg/kg group 27.72 ± 0.73, in the group with 100 mg/kg of tartrazine 32.74 ± 0.55, in the TAR 10 + CUR 20 mg/kg group, 23.78 ± 0.36, and in the TAR 100 + CUR 20 mg/kg group, 26.06 ± 0.67 was found. Table 2 presents the results of DNA damage in liver tissue. We observed an increase in the tail DNA ratio as the tartrazine dosage increased, and a decrease in the tail DNA level in the groups treated with curcumin. We visualized the analysis results using fluorescence microscopy to observe DNA damage, and we show the images in the comet assay results in Figure 1. Approximately 67% of the liver consists of liver cells called ‘hepatocytes’ (parenchyma cells). In addition, there are cell types such as bile duct epithelial cells, Ito cells, Kupffer cells and fibroblasts, which make up 37% of the liver tissue. In the comet assay, approximately 0.5 g of the whole liver tissue is taken and studied. A specific cell is not studied.

**TABLE 2:** Assessment of deoxyribonucleic acid impairment within hepatic tissue (Mean ± SEM).

Parameters	Control	TAR‐10 mg	TAR‐100 mg	TAR‐10 + CUR‐20 mg	TAR‐100 + CUR‐20 mg
Head length (μm)	131.40 ± 2.82	175.08 ± 5.02^a^	191.88 ± 6.29^a,b^	199.40 ± 6.80^b,c^	218.76 ± 7.42^c^
Tail length (μm)	29.19 ± 1.12	216.38 ± 13.72^a,b^	411.80 ± 33.73	253.16 ± 22.01^a^	238.34 ± 16.10^b^
Comet length (μm)	160.58 ± 3.45	387.86 ± 15.88^a,b^	600.04 ± 36.85	452.56 ± 25.45^a,c^	457.10 ± 18.19^b,c^
Percent head DNA	96.12 ± 0.12	72.10 ± 0.68^a^	67.26 ± 0.55	76.20 ± 0.36	73.94 ± 0.67^a^
Percent tail DNA	3.88 ± 0.12	27.72 ± 0.73^a^	32.74 ± 0.55	23.78 ± 0.36	26.06 ± 0.67^a^
Tail moment	1.34 ± 0.06	77.40 ± 8.36^a^	234.90 ± 28.10	94.96 ± 13.15^a^	84.78 ± 10.19
OTM	2.42 ± 0.12	44.58 ± 3.62^a^	132.32 ± 15.07	55.46 ± 5.98^a,b^	53.74 ± 5.12^b^

*Note:* Statistically significant disparities were observed among the groups across all measured parameters (*p* < 0.001). In the comparative analysis of the binary groups, the Student's *t*‐test was utilized. Parameters exhibiting statistically significant differences between the two groups were denoted with identical letters. The ANOVA (Analysis of Variance) test was employed in the statistical evaluation.

**FIGURE 2:**
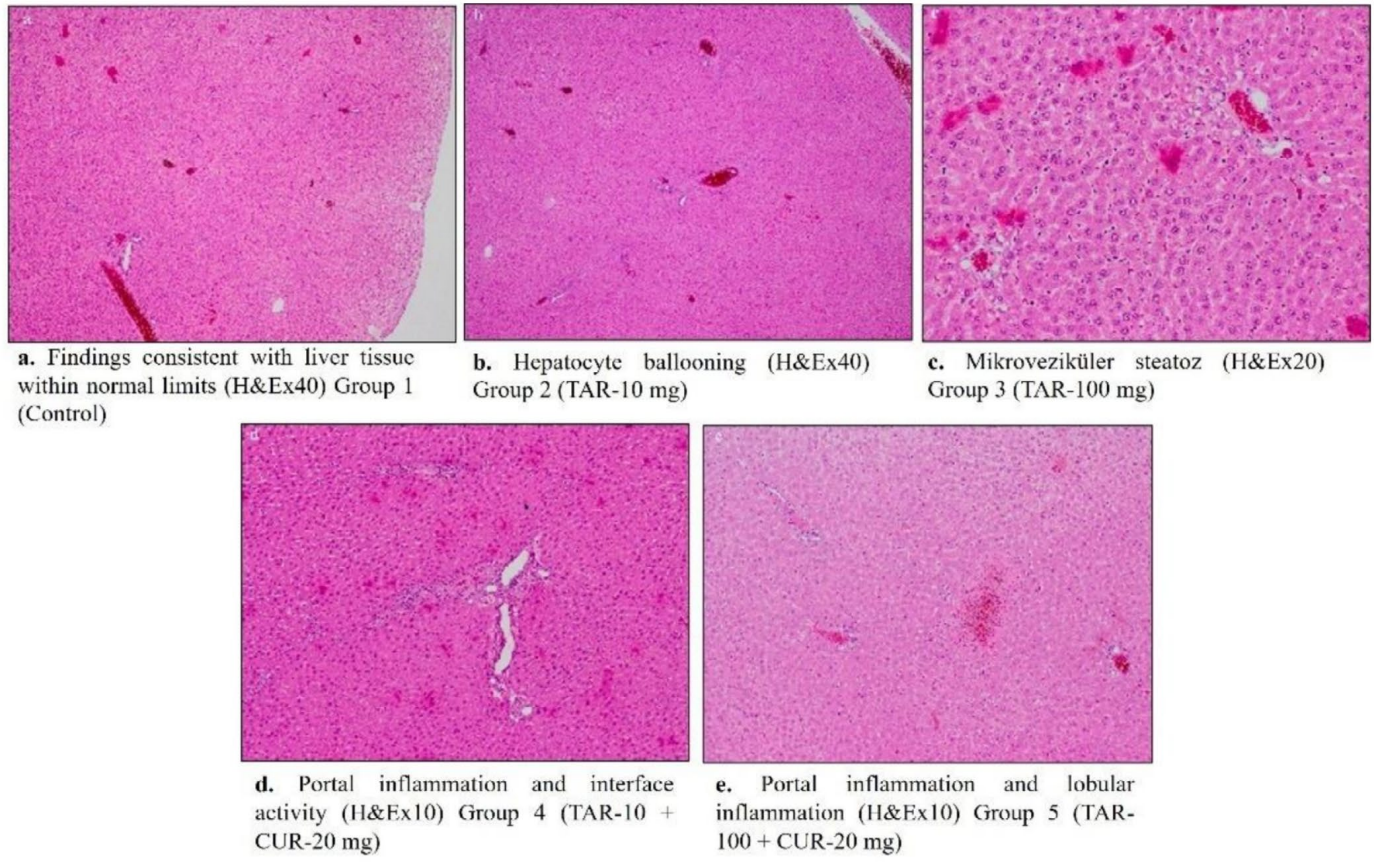
Histopathological examination of liver tissue in all experimental groups.

Figure 2 presents the histopathological results. Our findings in the control group were consistent with normal liver tissue. Group 2 (TAR 10 mg/kg) showed hepatocyte ballooning; Group 3 (TAR 100 mg/kg) displayed microvesicular steatosis and lobular inflammation; Group 4 (TAR 10 + CUR 20 mg/kg) showed portal inflammation and interface activity; and Group 5 (TAR 100 + CUR 20 mg/kg) showed lobular inflammation, portal inflammation, interface activity, and the presence of eosinophils in the portal area.

**FIGURE 3:**
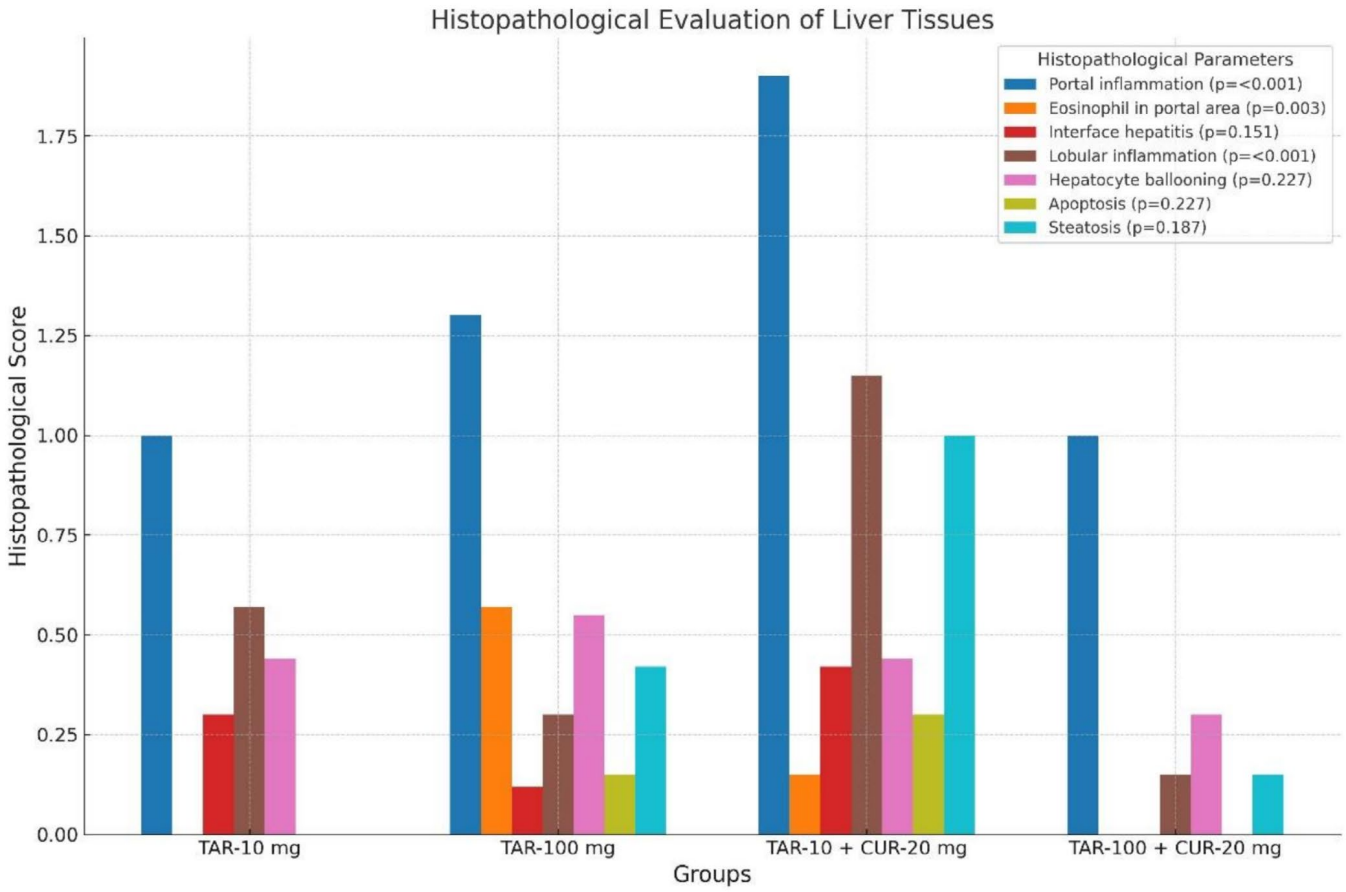
Histopathological evaluation of liver tissue (control group excluded).

Figure 3 presents the histopathological damage scores of liver tissue. The statistical analysis between the groups revealed a statistically significant difference in the parameters of portal inflammation, eosinophils in the portal area, and lobular inflammation, with a *p*‐value under 0.05. The scoring data showed no significant change in interface hepatitis, hepatocyte ballooning, apoptosis, and steatosis (*p* > 0.05). However, the scoring observation data showed no significant changes, and the low averages and quartile values (25th–75th percentiles) prevented statistical analysis for the parameters of Mallory‐Denk bodies, hepatocyte mitosis, bile duct epithelium damage, ductal proliferation, ductile reaction, bilirubin stasis, kolat internship, sinusoidal dilation, and venous congestion.

## 4 Discussion

In the literature, there are conflicting data regarding the toxic effects of tartrazine. In the conducted study, an increase in AST and ALT values was found in the group given tartrazine. (Ashfaq et al. 2023). In another study (Amin et al. 2023), tartrazine altered biochemical and antioxidant levels, resulting in higher levels of AST, ALT, ALP, GGT, AFP, and total bilirubin in the serum of the tartrazine groups (75 mg/kg). These results indicate that the application of food dyes can lead to an increase in free radical levels, ROS production, and a decrease in antioxidant mechanisms. (Albasher et al. 2020). Another study observed an increase in serum AST and ALT levels in the experimental group following 60 days of oral tartrazine (200 mg/kg bw) administration compared to the control group. In the oxidative stress parameter SOD, a decrease was observed compared to the control group (Ali et al. 2016). In the group given tartrazine (56 days, 300 mg/kg, male Wistar rats), induced tissue samples showed necrosis, fatty degeneration, chromatin condensation (pyknosis), disruption of regular hepatic cells, hydropic degeneration of cells, altered lobular morphology, and nuclear fragmentation in some areas. (Hanna et al. 2023). Tartrazine causes oxidative effects, biochemical profiles, and liver damage, according to a study investigating its cytotoxic effects (Amchova et al. 2024). However, it is stated that low‐dose curcumin regulates the serum AFP and lipid irregularities caused by the intake of tartrazine (El‐Desoky et al. 2022). In a study conducted on human leukocyte cell lines, it was reported that even at very low doses, tartrazine (70 μg mL^−1^) caused DNA damage (Floriano et al. 2018). In a long‐term treatment study with tartrazine (7.5 mg/kg), it was observed that serum AST, ALT, and ALP levels significantly increased compared to the control group after 90 days of exposure (Elekima et al. 2019). Our study revealed that the administration of high‐dose (TAR 100 mg/kg) tartrazine led to an increase in AST and ALT levels compared to the control group. This finding is consistent with previous research and may be due to oxidative stress. The group received low‐dose (TAR 10 mg/kg) tartrazine and curcumin, but curcumin had no significant effect. However, when the same group received high‐dose tartrazine and curcumin, curcumin lowered their serum AST and ALT levels and provided protection. We observed an increase in SOD levels as the tartrazine dose increased and a decrease in SOD levels when the groups received curcumin. We observed an increase in the serum level of LDH with high‐dose tartrazine and a decrease in the serum level with curcumin supplementation. We observed no significant change in the serum total bilirubin level compared to the control group. In the groups given low and high doses of tartrazine, serum ALP levels increased, while in both groups with curcumin, ALP levels decreased. The serum GGT level went up a lot in the high‐dose tartrazine (100 mg/kg) group. Adding curcumin to the medium brought the serum level back to that of the control group. The addition of curcumin did not significantly alter the amount of alpha‐fetoprotein in the serum. This suggests that tartrazine exposure immediately caused a protective cell response. The serum biochemistry data revealed a high degree of consistency with the literature regarding tartrazine intake. With this, biochemical findings may occur in response to short‐term metabolic changes and therapeutic agent exposure. A longer period is required for biochemical findings to change histopathological findings. It is also possible to see histopathological improvement in future longer‐term experiments. SOD is an antioxidant enzyme. It is a part of the oxidant‐antioxidant balance. Increased SOD activity basically indicates an increase in antioxidant capacity. However, it is also possible that an increase in reactive SOD was seen in response to oxidant effects in our study.

Abd Elaziz et al. (2024) found that animals given tartrazine by mouth for 30 days at doses of 25, 50, and 75 mg/kg showed mild to moderate changes in liver histopathology. Researchers found that low‐dose tartrazine treatment (90 days, 1.0 mg/kg/bw, and 2.0 mg/kg/bw) caused damage to cells in the liver, such as the death of hepatic cells and the emptying of vacuole cells (Shathi et al. 2023). Scientists say that curcumin (2 g) healed and protected the liver histology and related functional biomarkers of rats that were exposed to tartrazine (El‐Desoky et al. 2022). Researchers looked at liver tissue from rats that were given 200 mg/kg of tartrazine by mouth. They saw a lot of vacuolar degeneration in the liver parenchyma and major changes in the hepatoportal blood vessels and bile ducts in the portal area (Ali et al. 2016). Another study investigated the long‐term effects of tartrazine exposure. Under a microscope, they examined the liver tissue and discovered damaged structures. While looking at the liver through a microscope, they saw swollen hepatocytes, empty spaces, missing hepatic plates, colored Kupffer cells in the sinusoids, lobular boundaries that weren't lined up right, groups of swollen hepatocytes, loss of nuclear content in hepatocytes (pyknosis), hydrostatic breakdown of the central vein, and fatty materials around the central vein (Elekima et al. 2019). Our study revealed that tartrazine, in line with previous research, has the potential to induce certain histological changes in liver tissue during histopathological examinations. We saw that taking tartrazine can lead to hepatocyte ballooning, microvesicular steatosis, and lobular inflammation. On the other hand, taking curcumin led to degenerations like lobular inflammation, portal inflammation, interface activity, and the presence of eosinophils in the portal area. This situation indicates that curcumin does not have a protective effect on histopathology.

Researchers who used tartrazine (7.5–10 mg/kg) found changes in the liver's histology, such as swollen central veins, enlarged sinusoids, dying and darkly stained hepatic cells that are contracted, vacuolation, and swollen portal veins filled with lymphocytes. Abd Elaziz et al. (2024) found that compared to the control group, there was an increase in the number of inflammatory cells, the size of the central vein, the number of hepatocytes that were dying, and the congestion area in the mother's liver. Researchers have reported that Sunset Yellow (SY) increases the number of degenerative cells in the liver tissues of Swiss albino rats (Şensoy 2024). We performed histopathological scoring of liver tissue sections in our study. All groups showed statistically significant portal inflammation, lobular inflammation, and the eosinophil parameter in the portal area. We detected no significant changes among the groups for the other parameters. We observed no significant differences in the scoring analysis for interface hepatitis, hepatocyte ballooning, apoptosis, and steatosis. Due to insufficient data for scoring, we observed no significant changes in our evaluation of scoring parameters such as Mallory‐Denk bodies, hepatocyte mitosis, bile duct epithelial damage, duct proliferation, ductal reaction, bilirubin stasis, cholate retention, sinusoidal dilation, and venous congestion. The literature has detected similar changes.

Researchers have investigated the genotoxic effects of tartrazine exposure on rat leukocytes. The study observed a significant increase in the tail DNA ratio compared to the control group. Researchers have observed DNA damage in both kidney and liver tissues using the comet assay technique. (Khayyat et al. 2017). Another similar study reported that tartrazine, when administered at very low concentrations (0.25–64.0 mM) to human lymphocytes, could have a significant genotoxic effect at all concentrations. (Soares et al. 2015). Sakaki and colleagues investigated the genotoxic effects of food supplements in doses of 10–100 mg/kg in eight mouse organs using the comet assay method. They indicated that the doses given in the study would cause DNA damage in gastrointestinal organs. (Sasaki et al. 2002). In our study, we observed that tartrazine intake at doses of 10 and 100 mg/kg caused DNA damage compared to the control group, and curcumin supplementation could repair this damage. The literature reveals a scarcity of studies exploring tartrazine's potential effects on liver tissue through the comet method. With this, all antioxidants have some damage to DNA and tissue. Unfortunately, when antioxidants are applied to healthy cells, they cause a toxic effect on the cells, while they also cause positive effects on damaged cells.

## 5 Conclusion

We administered tartrazine orally to rats in our study at doses above the daily acceptable limit. We administered tartrazine at doses of 10 mg/kg and 100 mg/kg daily. In our twenty‐one‐day study, it was observed that tartrazine caused DNA damage, led to changes in biochemical parameters related to liver tissue, and caused histopathological changes in liver tissue, and that the supplemented curcumin could have a protective effect. It is evident that tartrazine, an azo dye, would have a toxicological effect. Looking at scientific study reports, it appears that the food additives and colorants currently in use require a more comprehensive evaluation. Biochemical findings may occur in response to short‐term metabolic changes and therapeutic agent exposure. A longer period is required for biochemical findings to change histopathological findings. It is also possible to see histopathological improvement in future longer‐term experiments.

## Author Contributions

**Metin Varlı:** conceptualization (equal), investigation (equal), methodology (equal), resources (equal), supervision (equal), writing – original draft (equal), writing – review and editing (equal). **Mehmet Cihan Yavaş:** conceptualization (equal), data curation (equal), formal analysis (equal), investigation (equal), methodology (equal), resources (equal), supervision (equal), validation (equal), writing – original draft (equal), writing – review and editing (equal). **Fazile Cantürk Tan:** data curation (equal), formal analysis (equal), investigation (equal), resources (equal), writing – original draft (equal), writing – review and editing (equal). **Kardelen Tur:** data curation (equal), formal analysis (equal), investigation (equal), resources (equal), writing – original draft (equal), writing – review and editing (equal). **Güldidar Basmacı:** data curation (equal), formal analysis (equal), writing – original draft (equal), writing – review and editing (equal).

## Ethics Statement

Ethical permissions were obtained from Dicle University Health Sciences Research and Application Center Animal Experiments Local Ethics Committee (DUHADEK) (25/09/2024, Decision no: 01, Meeting number: 12).

## Conflicts of Interest

The authors declare no conflicts of interest.

## Data Availability

Our data is not open, but researchers who wish to access the data set can request it through the relevant ethics committee or by emailing the corresponding author.
